# Epidemiology and surveillance of human animal-bite injuries and rabies post-exposure prophylaxis, in selected counties in Kenya, 2011–2016

**DOI:** 10.1186/s12889-018-5888-5

**Published:** 2018-08-09

**Authors:** Jeremiah Ngurimu Ngugi, Alfred Kilango Maza, Owiti Jack Omolo, Mark Obonyo

**Affiliations:** 1 Kenya Field epidemiology and Laboratory Training Program, Nairobi, Kenya; 2 Department of Veterinary Services, County Government of Taita Taveta, Taveta, Kenya; 3 Regional Veterinary Investigation Laboratory, Mariakani, Kenya; 4 Department of Veterinary Services, County Government of Kilifi, Kilifi, Kenya; 5 Ministry of Agriculture, Livestock and Fisheries, Directorate of Veterinary Services, Nairobi, Kenya

**Keywords:** Rabies, Epidemiology, Post-exposure prophylaxis, Surveillance

## Abstract

**Background:**

Human animal-bite injuries are a serious public health problem due to associated risk for rabies virus exposure. Animal-bite injuries especially dog bites are useful indicators for assessing the risk of rabies virus transmission and need for rabies post exposure prophylaxis (PEP). Understanding the epidemiology and surveillance of animal bites and rabies post-exposure prophylaxis is critical in implementing Kenya’s national rabies elimination strategy. We aimed to describe the incidence of human animal-bite injuries, patient/biting animal characteristics, uptake of rabies PEP and factors associated with animal bite incidents.

**Methods:**

We reviewed animal bite records from outpatient and anti-rabies vaccine (ARV) registers of 17 health facilities from five counties. An animal bite was defined as an entry of an animal bite of the class mammal including humans in registers in a person of any age from January 2011 to December 2016. We collected demographic and information on PEP uptake. We calculated descriptive statistics, odds ratios (OR) and 95% confidence interval (CI) to examine factors associated with being an animal bite case-patient. We also calculated incidence of animal bites using health facility catchment population for year 2016 as the denominator.

**Results:**

We analyzed 7307 records. The median age was 22 years (IQR = 31 years); there were 4019 (55%) male and age < 15 years were 2607 (37%). Dogs accounted for 6720 (93%) of bites of which 78% were owned free-roaming dogs. Of the 5674 (88%) cases that received rabies PEP, 2247 (40%) got at least three-doses. The median time from bite to seeking medical care was 2 days (IQR = 4 days). Being bitten on the head/face (OR = 5.8; CI: 3.3–10.2); being bitten by owned free-roaming dog (OR = 1.7; CI: 1.5–1.9) and being male (OR = 1.4; CI: 1.3–1.5) were significantly associated with being an animal-bite case-patient. Being male, being bitten on head/face and being bitten by owned free-roaming dog remained independently associated with being an animal bite case-patient at multivariable logistic regression. Bite-incidence was 289 bites /100,000 persons among all counties.

**Conclusion:**

Preventing dog bites would most effectively reduce bite injuries by improving public health education among children below 15 years, encouraging early PEP initiation and completion, development and implementation of responsible dog ownership and animal behaviour educational programmes as well as improving human and veterinary health linkages.

**Electronic supplementary material:**

The online version of this article (10.1186/s12889-018-5888-5) contains supplementary material, which is available to authorized users.

## Background

Human animal-bite injuries are a serious public health problem owing to the associated risk of rabies virus exposure especially in rabies endemic countries [1–4]. Approximately 85 to 90% of human animal-bite injuries are caused by dogs, 5 to 10% by cats and 2 to 3% by humans and rodents [2]. In low income countries, several studies have demonstrated that dogs account for 76 to 94% of animal-bite injuries resulting into high prevalence of rabies and higher fatality rates due to poor access to anti-rabies post exposure treatment [5–7]. In Kenya, 146,362 (incidence 336 bites/100,000 persons) animal-bite injuries cases and 858 confirmed human rabies cases from owned free-roaming dogs have been documented between 2002 and 2012 [8, 9].

Rabies is the most significant public health concern following animal-bites’ injuries. Other public health concerns following animal-bites include the risk of sepsis of bite wounds, psychological trauma and high cost of seeking PEP for the bite victims [10–12]. Human mortality from canine rabies is estimated at 60,000 annually worldwide, with about 56% of the cases occurring in Asia and 43.6% in Africa, mostly in rural areas [8, 13, 14]. In Kenya, rabies is ranked among the top five priority zoonotic diseases and an estimated 1000 to 2000 cases of human rabies occur annually [8]. The human rabies burden in Kenya is thought to be underestimated due to factors limiting the utilization of rabies PEP including poor diagnostic capacity, inaccessibility of anti-rabies vaccine and poor knowledge and practices in management of animal-bite injuries by affected persons [1, 8, 13]. Globally, more than 15 million people receive a post-bite vaccination costing about US$ 1.7 billion annually [3, 14]. In Africa, approximately 200,000 persons obtains anti-rabies PEP annually following an incidence of animal bite to prevent them from developing rabies [7]. Studies have estimated the average cost of rabies post-exposure prophylaxis (PEP) at US$ 40 in Africa, and US$ 49 in Asia resulting into catastrophic financial burden on affected families whose average daily income is estimated at around US$ 1–2 per person [13–15]. A study in Tanzania found the cost of PEP per animal bite victim to be over US$ 70 [6], while another study in Kenya found the cost of a single dose of PEP to range between US$ 8 to US$ 120 [1]. Kenya’s Zoonotic Disease Unit (ZDU) estimated the direct medical cost associated with a complete regime of PEP to be about US$ 85 per person [8].

Kenya is implementing a national human rabies elimination strategy with a target of achieving zero human deaths from dog mediated rabies by 2030. Using three pronged approach, the strategy aims to reduce the disease risk through sustained mass dog vaccination, dog population management, pre and post-exposure prophylaxis in humans and public education among animal/human health workers and the community. With an estimated 5 million dogs in Kenya, 80% of which have known owners, mass vaccination of owned dogs would cover a substantial population of dogs and can successfully eliminate canine mediated rabies [8]. Animal-bite injuries have been used as a proxy to assess the risk of transmission of rabies virus from animals to humans and determine need for PEP [16]. Surveillance data of animal-bite injuries can also be used as a proxy to estimate an area or region specific rabies burden, thus prioritizing improved rabies surveillance and control [16]. Further, this data can help in understanding patient as well as biting animal characteristics and spatial-temporal distribution of animal-bite injuries [13, 16]. These are useful indictors in monitoring the success in implementation of the rabies elimination strategy. We therefore aimed to characterize animal bite cases, biting animals’ characteristics, and PEP treatment and evaluate the exposure factors associated with the animal-bite injuries.

## Methods

### Study site

**Fig. 1:**
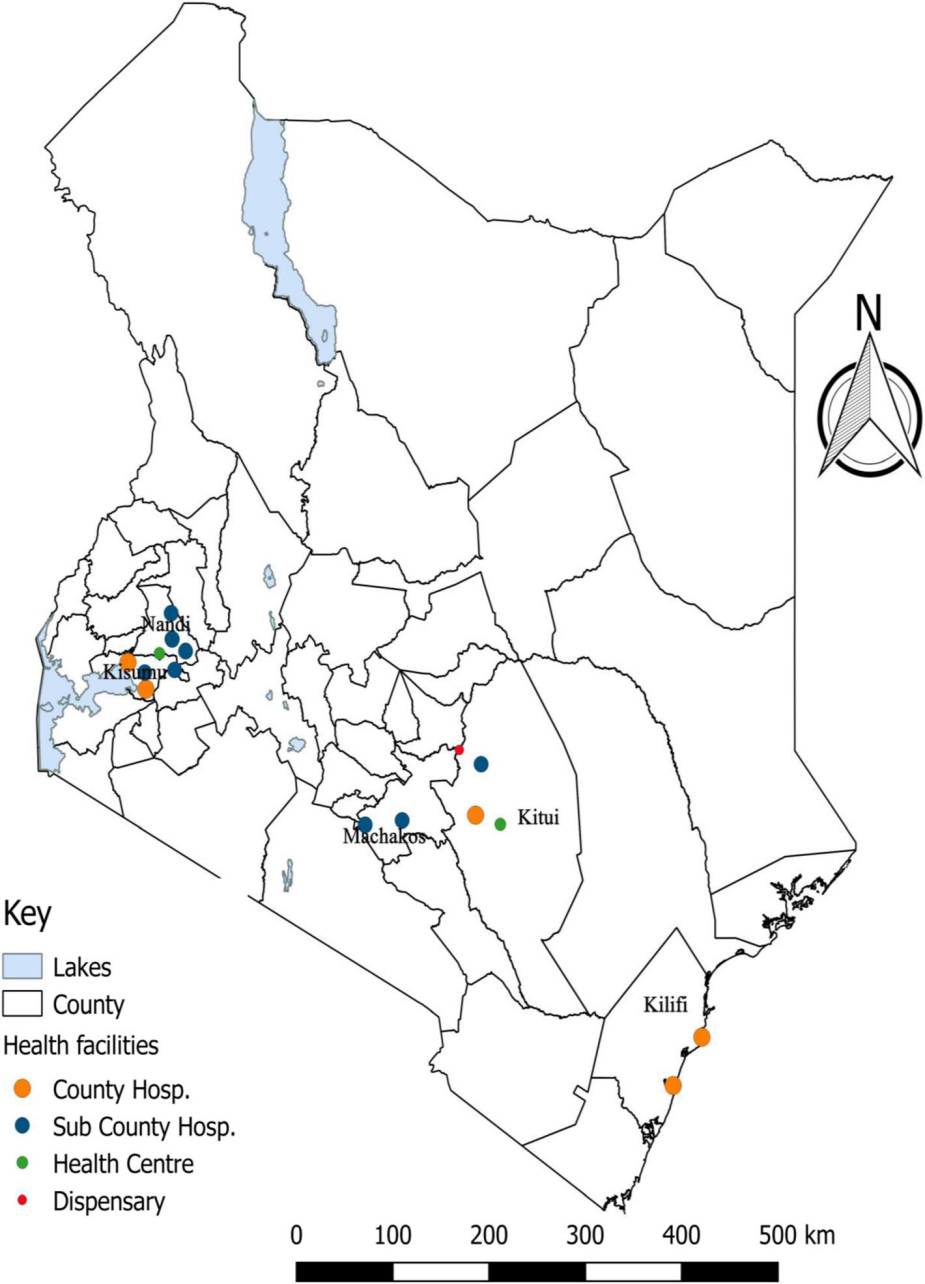
Study area showing distribution of the study sites – Kenyan Map. Counties Sampled (Map developed by Author using QGIS Version 2.18.10) with geographical data was obtained from https://africaopendata.org/dataset/kenya-counties-shapefile and data on geo - coordinates and category of health facilities was obtained from Kenya Master Health facility list http://kmhfl.health.go.ke/

The study was conducted in five counties namely, Machakos and Kitui counties in lower eastern region; Kisumu County in Lake Victoria basin; Nandi County in Central rift valley and Kilifi County in the coastal region. We selected a total of 17 government health facilities offering PEP in the five counties comprising of County referral hospitals, Sub-county hospitals, health centres and peripheral health facilities located in both urban and rural settings (Fig. 1).

### Retrospective review of registers

We reviewed out-patient and anti-rabies vaccine registers in the selected health facilities from January 2011 to December 2016. An animal bite was defined as any entry of an animal bite of the class mammal including humans in the registers for a person of any age during the review period. We defined owned dog as dogs that are always confined or partly confined and spend part of their time at home and part of their time roaming unsupervised outside of the owner’s property and the bite injury victim reported they knew the owner. Unowned dogs were free roaming dogs without a known owner. We used a standardized data abstraction tool to collect the following information: date of bite, date seen at health facility, age, sex, residence, species of biting animal, type of biting animal (for only cats and dogs, classified as whether owned or unowned), site of bite, whether PEP was given and number of PEP doses administered Additional file 1. Kenya uses the WHO recommended five intramuscular doses of rabies PEP on days 0, 3,7,14 and 28 and RIG infiltration for category III bites [1]. However RIG is rarely offered to animal bites victims due to the high cost.

### Data management

We managed data using Microsoft Excel 2016 (Microsoft, Seattle, WA, USA), and analyzed using Epi Info version 7 (CDC, Atlanta, GA, USA). We calculated proportions for categorical variables and means and medians for continuous variables. We performed bivariate and multivariable unconditional logistic regression analysis and calculated odds ratios (OR), adjusted odds ratio (AOR) and 95% confidence interval (CI) using age-group < 15 years and 15+ years as an dependent variable and other variables such as sex of bite victim, species of biting animal, type of biting animal, site of bite, whether PEP was given or not and number of doses of PEP given as independent variables to determine factors associated with being a bite patient. We also calculated incidence of animal bites per 100,000 persons using the health facility catchment population for the year 2016 as the denominator as well as rate ratio calculated as conditional maximum likelihood estimate (CMLE) with 95% CI for sex, age-group (stratified as < 15 and 15+ years) and Counties and we considered *p*-value ≤0.05 to be statistically significant.

## Results

### Demographic characteristics and site of bite

Among 7307 records analyzed, 7201 (98.6%) had age recorded. The median age was 22 years (Q1 = 11, Q3 = 42 years) and age group 5–14 years old were 2138 (30%) and 469 (7%) were children under five years. Of 7305 (99.9%) records where sex was captured, 4019 (55%) were male. A total of 3345 (66%) lower extremities (leg/thigh), 987 (20%) on arms and 210 (4%) in the head/face (Table 1).

**Table 1:** Demographic characteristics, bite location, biting animal and PEP treatment among animal bite victims distributed by County, 2011–2016

Variable	All cases n (%)	Kilifi (*n* = 1931) n (%)	Kisumu (*n* = 2071) n (%)	Kitui (*n* = 1266) n (%)	Machakos (*n* = 295) n (%)	Nandi (*n* = 1762) n (%)
Sex						
Female	3286 (45)	822 (43)	872 (42)	604 (48)	144 (45)	844 (48)
Male	4019 (55)	1091 (57)	1199 (58)	662 (52)	151 (51)	916 (52)
Age group						
< 5	469 (6)	91 (5)	50 (2)	100 (9)	25 (9)	203 (11)
5–14	2138 (30)	686 (36)	231 (11)	520 (45)	116 (39)	585 (33)
15–24	1236 (17)	344 (18)	391 (19)	165 (14)	42 (14)	293 (17)
24–39	1381 (19)	354 (19)	462 (22)	169 (15)	52 (18)	341 (20)
40–59	1464 (20)	298 (16)	735 (36)	135 (12)	46 (16)	248 (14)
60+	513 (7)	140 (7)	202 (10)	74 (6)	14 (5)	83 (5)
Bite location						
Leg/Thigh	3345 (66)	681 (64)	1343 (65)	346 (57)	204 (69)	771 (77)
Arm	987 (20)	281 (26)	288 (14)	186 (31)	52 (18)	180 (18)
Trunk	299 (6)	84 (8)	172 (8)	26 (4)	10 (3)	7 (0.7)
Head/Face	210 (4)	24 (2)	127 (6)	22 (4)	11 (4)	26 (3)
Buttock/Groin	197 (4)	Nil	141 (7)	25 (4)	17 (6)	14 (0.7)
PEP administered						
Yes	5674 (88)	1022 (96)	1774 (86)	844 (67)	295 (100)	1739 (99)
No	789 (12)	48 (5)	297 (14)	422 (33)	Nil	22 (1)
No of PEP doses						
1	2008 (35)	145 (14)	571 (32)	286 (34)	58 (20)	948 (55)
2	1411 (25)	109 (11)	812 (46)	146 (17)	39 (13)	305 (18)
3	1100 (19)	107 (11)	365 (21)	115 (14)	120 (41)	393 (23)
4	377 (7)	166 (16)	23 (1)	133 (16)	15 (5)	40 (2)
5	770 (14)	495 (48)	Nil	162 (19)	63 (21)	50 (3)
Type of biting animal						
Owned dog ^a^	5208 (72)	1575 (82)	1058 (51)	990 (78)	270 (92)	1315 (76)
Unowned Dog	1512 (21)	324 (17)	899 (43)	170 (13)	16 (5)	103 (6)
Owned Cat	380 (5)	14 (0.7)	63 (3.0)	24 (2)	3 (1)	276 (16)
Livestock ^b^	77 (1)	Nil	10 (0.4)	56 (4)	1 (0.3)	Nil
Human	47 (0.7)	Nil	24 (1)	11 (0.9)	Nil	12 (0.7)
Unowned Cat	34 (0.5)	Nil	17 (0.8)	15 (1)	1 (0.3)	1 (0.1)
Others ^c^	12 (0.2)	Nil	Nil	Nil	4 (0.7)	7 (0.4)

^a^Owned dog refers to dogs that are partly confined and spend part of their time roaming unsupervised outside the owner’s property while unowned dogs refers free roaming dogs without a known owner

^b^Livestock includes donkey and pig.

^c^others include monkeys, rats, hyena and other wild animals.

Overall, the population adjusted human animal-bite injuries incidence was 289 per 100,000 persons with the highest incidence reported in Kilifi at 302 per 100,000 and lowest in Machakos at 121 per 100,000 persons. The age-group < 15 years had a higher incidence in Kitui, Machakos and Nandi counties while males had a higher incidence across all the counties. There was a statistical significant difference in bite incidence for sex, age-group and Counties (*p*-value ≤0.05) (Table 2).

**Table 2:** Population adjusted human animal-bite injuries incidence per 100,000 persons by Sex, Age group and County, 2011–2016

Variable	Catchment pop (Yr. 2016)	Bite cases	Average cases per year	Bite Incidence/100,000 persons	CMLE Rate Ratio	*P*-value
Sex						
Male	243,029	4019	804	331	1.4 (1.2, 1.5)	< 0.0001
Female	272,094	3286	657	242	Reference	
Age group						
< 15 years	215,139	2607	521	242	0.8 (0.7, 0.9)	< 0.0001
15+ years	289,984	4594	919	317	Reference	
Counties						
Kilifi	126,843	1913	383	302	0.8 (0.7, 1.0)	0.0157
Kitui	93,485	1266	253	271	0.8 (0.6, 0.9)	0.0002
Machakos	48,684	295	59	121	0.3 (0.3, 0.5)	< 0.001
Nandi	120,612	1762	353	292	0.8 (0.7, 0.9)	0.0051
Overall	505,124	7307	1461	289	0.8 (0.7, 0.9)	0.0002
Kisumu	115,500	2071	414	359	Reference	

### Delay in seeking treatment and PEP administration

**Fig. 2:**
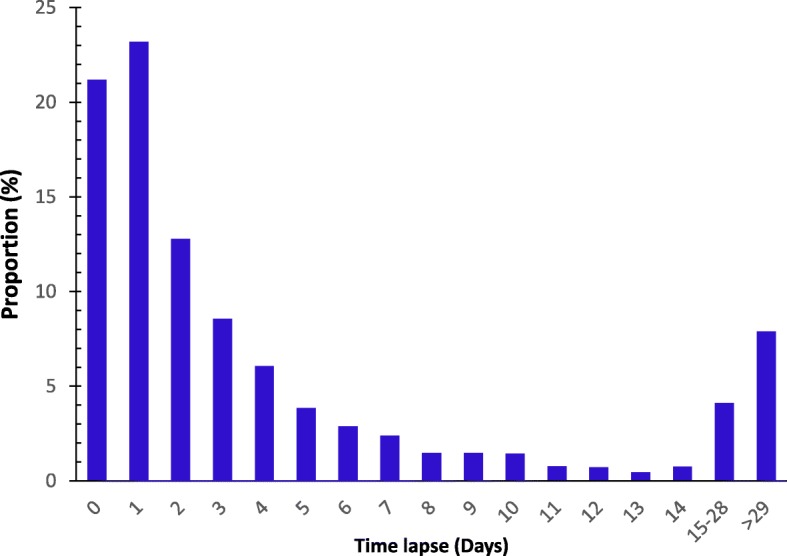
No of cases (%) by time in days between contact of bite and visiting a health facility (n-3409). Proportion of cases (%) by period (days) that reported to a health facility after an animal bite from 0 to 29 days

The median number of days between contact with biting animal and presenting at a health facility was 2 days (Q1 = 1, Q3 = 6 days). A total of 1989 (57%) of the animal-bite victims accessed a health facility within the first two days after the bite (Fig. 2). Among the counties, 295 (100%) bite cases in Machakos, 1739 (99%) in Nandi and 844 (67%) in Kitui were started on rabies PEP. At-least 2247 (40%) of the cases received three doses of PEP with 768 (75%) in Kilifi, 198 (67%) in Machakos, 410 (49%) in Kitui, 483 (28%) in Nandi, and 388 (22%) in Kisumu (Table 1).

### Biting animal characteristics

Of the 7270 (99.5%) cases whose records on biting animal were recorded, 5208 (72%) bites were inflicted by owned-dogs, with 270 (92%) in Machakos, 1575 (82%) in Kilifi, 1315 (76%) in Nandi, 990 (78%) in Kitui, and 1058 (51%) in Kisumu. Un-owned dogs were responsible for 1512 (21%) of the bites distributed with 899 (43%) recorded in Kisumu, 324 (17%) in Kilifi, 170 (13%) in Kitui 16 (5%) in Machakos and 103 (6%) Nandi (Table 1).

### Exposure factors

Children below 15 years had increased chances (OR = 1.7, 95% CI: 1.5, 1.9) of being bitten by owned-dogs compared to those aged above 15 years. However unowned dogs had lower chances (OR = 0.6, 95% CI 0.5, 0.7) of causing bite injuries in children below 15 year olds compared to children above 15 years old. Male below 15 years had increased chances of suffering animal-bite injuries compared to female of the same age category (OR = 1.4, 95% CI 1.3, 1.5) and children below the age of 15 years had increased chances (OR = 5.8 (95% CI 3.3, 10.2) of having bites inflicted on the head and face compared to those aged above 15 years (Table 3). On multivariable unconditional logistic regression analysis: being male (aOR 1.5 (95% CI 1.3, 1.7), having bite injuries on the head/face (aOR 1.8 (95% CI 1.3, 2.6) and being bitten by owned dog (aOR 1.2 (95% CI 1.1, 1.3) remained significantly associated with the being animal-bite.

**Table 3:** Bivariate and multivariable logistic regression analysis for exposure factors associated with animal-bite in selected counties, Kenya, 2011–2016

Variable	Young (< 15 years) (2351) n (%)	Adult (15+ years) (*n* = 4382) n (%)	OR (95% CI)	Adjusted Odds Ratio (AOR)
Biting animal				
Owned Dog ^a^	2011 (78)	3094 (68)	1.7 (1.5, 1.9)	1.2 (1.1, 1.3)
Unowned Dog	403 (16)	1109 (24)	0.6 (0.5, 0.7)	NS
Cat	152 (6)	262 (6)	1 (0.8, 1.3)	NS
Human	3 (0.1)	43 (0.9)	0.1 (0.02, 0,38)	NS
Livestock ^b^	21 (0.8)	55 (1)	0.7 (0.43, 1.11)	NS
Others ^c^	2 (0.1)	10 (0.2)	0.4 (0.04, 1.66)	NS
Sex				
Male	1573 (60)	2400 (52)	1.4 (1.3, 1.5)	1.5 (1.3, 1.7)
Female	1034 (40)	2193 (48)	Ref	
Bite Site				
Leg/Thigh	775 (60)	1173 (74)	0.5 (0.5, 0.6)	NS
Arm	323 (25)	344 (22)	1.2 (1, 1.5)	NS
Trunk	83 (7)	39 (3)	2.8 (1.9, 4.1)	NS
Head/Face	67 (5)	15 (1)	5.8 (3.3, 10.2)	1.8 (1.3, 2.6)
Buttock/Groin	36 (3)	17 (1)	2.7 (1.5, 4.8)	NS
PEP				
Yes	1942 (84)	3629 (90)	0.6 (0.54, 0.72)	0.3 (0.2, 0.3)
No	364 (16)	423 (10)	Ref	
PEP Doses				
≥ 3 doses	875 (40)	1293 (60)	1.5 (1.3, 1.7)	NS
< 3 dose	1062 (31)	2333 (69)	Ref	
Counties				
Kilifi	777 (30)	1136 (25)	4.4 (3.7, 5.1)	5.2 (4.3, 6.3)
Kitui	620 (24)	549 (12)	7.2 (6.1, 8.5)	6.4 (5.3, 7.6)
Machakos	141 (5)	154 (3)	5.8 (4.5, 7.6)	6.1 (5.0, 8.0)
Nandi	788 (30)	965 (21)	5.2 (4.5, 6.1)	5.7 (4.8, 6.8)
Kisumu	281 (11)	1790 (39)	Ref	

*NS* Not significant

^a^Owned dog refers to dogs that are partly confined and spend part of their time roaming unsupervised outside the owner’s property while unowned dogs refers free roaming dogs without a known owner.

^b^Livestock includes donkey and pig.

^c^others include monkeys, rats, hyena and other wild animals.

## Discussion

This study demonstrates that animal-bite injuries remain an important cause of morbidity and reason for visiting health facilities in Kenya. This is important since bite injuries is the main mode of transmission of human rabies [3, 6–8, 10]. This study also identified high risk groups for animal-bite, culpable animals that are most likely to cause bites and exposure factors associated with animal-bite. This are useful information that may inform policy makers and implementers of the national human rabies elimination strategy in Kenya of ways they can design possible interventions with most impact to reduce human rabies.

Owned dogs were implicated in most animal-bite injuries. This is consistent with other studies that have implicated owned dogs in most of bite injury incidents [1, 16]. In Kenya and many other rabies-endemic countries dog owners allow their dogs to roam freely [17, 18], which increases an animals’ risk for rabies virus exposure and transmission to other animals and also humans. In Kenya, a study conducted in Machakos County, reported that only 29% of the owned dogs were vaccinated against rabies. Similar reports by ZDU-Kenya indicated that only 23% of owned dogs were routinely vaccinated in Makueni county [17, 18] while another study estimated that dog vaccination coverage in Kenya was 0.5% [3]. In tandem with the increased use of dogs as companion and guard animals in Kenya, the number of dog bites is likely to increase and as a result there will likely be an increase in rabies transmission especially in rural areas where dogs are not leashed and move freely thereby increasing the risk of exposure to rabies [16–19].

While the most frequently bitten body part was the limbs, children were predisposed to bites on the head and face due to their stature [20]. Animals bites to the head and face are especially risky due proximity to the central nervous system and clinical progression to rabies is highly likely if the bite occurs from a rabid animal. It is therefore advisable that such children should receive immediate PEP and rabies immunoglobulin (RIG) treatment as soon as possible to prevent any possibilities of getting rabies. However in Kenya and most sub-Saharan Africa where rabies is endemic, this may not be possible due to several factors including distance from nearest health facility, availability of PEP and RIG in the health facilities and costs associated with obtaining PEP [1].

In this study, children below the age of 15 years comprised more than a third of the cases of animal-bite injuries. The same age group also had increased chances of suffering bite by owned dogs compared to unowned dogs. These findings are similar to previous studies that reported dog bites to be a major public health problem worldwide especially among male children below the age of 15 years [2, 4, 9, 13]. This is very critical in rabies elimination strategy using mass dog vaccinations since this age group are the ones who normally bring dogs for vaccinations (authors observation) and they are also critical group for targeting public health education to prevent rabies transmission following an incident of animal-bite including wound washing with soap and water, access to PEP and responsible dog ownership.

The PEP completion rates for those who got at least three doses varied across the counties. This is consistent with other studies where persons exposed to rabies did not receive the full course of rabies PEP treatment and often delayed starting the prophylactic treatment [4, 12, 21]. The low rabies PEP completion rates could be attributed to factors like the high cost of obtaining PEP, long distances to health facilities, multiple referrals, unavailability of rabies PEP in government health facilities among others [1]. WHO guidelines recommends use of rabies PEP and rabies immunoglobulin (RIG) as immediate critical first aid in-order to neutralize the virus before immunity from vaccination develops [9, 15, 21]. Therefore, collaborations between the human and animal health sectors using an integrated one health approach has been demonstrated to significantly improve the number of people starting and completing PEP while reducing the unnecessary use of rabies PEP for non-rabies exposures [22].

## Limitations

The animal bite surveillance in Kenya is passive, as a result the main limitations of this study was incomplete data from the data sources (Outpatient and anti-rabies registers). Further, this study describes patients that seek care in health facilities and therefore miss a critical population that don’t seek medical attention. The rabies vaccination status of the animal causing the injury was also unknown, the circumstances of the bite incident and information on whether the biting animals were suspected of being rabid or not was not available. All these limited the scope of analysis for this study.

## Conclusions

Animal-bite injuries from owned dogs remain a major public health problem in Kenya and biggest contributor to rabies exposures especially in male and children below 15 years. To achieve the goal of eliminating dog mediated human rabies by 2030 as enshrined in the Kenya rabies elimination strategy blue print, sustained mass vaccination of owned dogs will be critical. Vaccination of the owned dogs against rabies has been documented to result in significant reduction in the incidence of human rabies and is cost-effective control strategy in the medium to long term [1, 7, 8]. However, other strategies outlined in the document like dog population management, pre and post-exposure prophylaxis in humans and public education among animal/human health workers and the community are also critical in achieving this goal. The success of the strategy will require participation and collaboration of every key stakeholder especially Country governments’ to allocate resources for mass dog vaccinations and educational sector in development of educational programs for children and dog owners regarding responsible dog ownership and animal behavior to reduce and prevent animal-bite cases. Strengthening Linkage and collaboration between the human and animal health sectors through the one health approach is critical for the success of the strategy.

## Additional file

Additional file 1: Kenya animal bites dataset. (XLSX 1570 kb)

## Abbreviations

AOR: Adjusted odds ratio
ARV: Anti Rabies Vaccine
CI: Confidence Interval
IQR: Interquartile Range
OR: Odds Ratio
PEP: Post exposure Prophylaxis
Q1: First Quartile
Q3: Third Quartile
RIG: Rabies Immunoglobulin
US$: US dollars
WHO: World Health Organisation
